# Ultra-Processed Food Consumption and Subclinical Cardiac Biomarkers: A Cross-Sectional Analysis of U.S. Adults in NHANES 2001–2004

**DOI:** 10.3390/nu17203294

**Published:** 2025-10-20

**Authors:** Jiahuan Helen He, Shutong Du, Valerie K. Sullivan, Lauren Bernard, Vanessa Garcia-Larsen, Eurídice Martínez-Steele, Ana Luiza Curi Hallal, Julia A. Wolfson, Mika Matsuzaki, Amelia S. Wallace, Mary R. Rooney, Michael Fang, John W. McEvoy, Elizabeth Selvin, Casey M. Rebholz

**Affiliations:** 1 Welch Center for Prevention, Epidemiology, and Clinical Research, Johns Hopkins University, Baltimore, MD 21205, USA; jhe75@jhmi.edu (J.H.H.); shutod@jhu.edu (S.D.); vsulliv5@jhmi.edu (V.K.S.); lauren.bernard@som.umaryland.edu (L.B.); awallace@jhu.edu (A.S.W.); mroone12@jhu.edu (M.R.R.); mfang9@jh.edu (M.F.); eselvin@jhu.edu (E.S.); 2 Department of Epidemiology, Johns Hopkins Bloomberg School of Public Health, Baltimore, MD 21205, USA; 3 University of Maryland School of Medicine, Baltimore, MD 21202, USA; 4 Program in Human Nutrition, Department of International Health, Johns Hopkins Bloomberg School of Public Health, Baltimore, MD 21205, USAjwolfso7@jhu.edu (J.A.W.); mmatsuz2@jhu.edu (M.M.); 5 Department of Nutrition, School of Public Health, University of São Paulo, São Paulo 01246-904, Brazil; emar_steele@hotmail.com; 6 Center for Epidemiological Studies in Health and Nutrition, University of São Paulo, São Paulo 01246-904, Brazil; 7 Departamento de Saúde Pública, Universidade Federal de Santa Catarina, Florianópolis 88040-900, Brazil; anacuri@gmail.com; 8 Department of Health Policy and Management, Johns Hopkins Bloomberg School of Public Health, Baltimore, MD 21202, USA; 9 National Institute for Prevention and Cardiovascular Health, School of Medicine, University of Galway, H91 FF68 Galway, Ireland; johnwilliam.mcevoy@nuigalway.ie

**Keywords:** cardiac troponin, NT-proBNP, subclinical myocardial stretch, ultra-processed food

## Abstract

Background/Objectives: Ultra-processed food consumption has been shown to be linked with clinical cardiovascular disease. This study aims to examine the associations of ultra-processed food consumption with biomarkers for subclinical-level myocardial damage [high-sensitivity cardiac troponin I and T (hs-cTnI and hs-cTnT)] and myocardial stretch (NT-proBNP) in U.S. adults. Methods: We used data from 6615 U.S. adults aged ≥20 years without prevalent cardiovascular disease from the National Health and Nutrition Examination Survey 2001–2004. We identified ultra-processed food by applying the Nova classification to dietary recall data, and we divided participants into quartiles based on their consumption, expressed as a proportion of total daily energy (%kcal) and gram intakes (%grams). We defined elevated cardiac biomarkers as hs-cTnI > 12 ng/L in men and >10 ng/L in women, hs-cTnT ≥ 14 ng/L for all participants, and NT-proBNP ≥ 125 pg/mL for age < 75 y and ≥450 pg/mL for age ≥ 75 y. We used multivariable logistic regression with adjustment for socio-demographic, total energy intake, behavioral, and clinical characteristics. Results: Higher ultra-processed food intake in %grams was associated with elevated NT-proBNP [odds ratio (OR) for quartile 4 vs. 1: 1.27, 95% CI: 1.00–1.61] when socio-demographic characteristics and total energy intake were adjusted for, but this was not the case with hs-cTnI or hs-cTnT. Further adjusting for clinical characteristics attenuated the association with NT-proBNP (OR: 1.26, 95% CI: 0.98, 1.61). There was no consistent association between ultra-processed food in %kcal and elevated NT-proBNP, hs-cTnT, or hs-cTnI. Conclusions: Ultra-processed food consumption is associated with subclinical myocardial stretch, a precursor to early heart failure. This supports the potential risks of subclinical cardiovascular disease associated with consuming ultra-processed food.

## 1. Introduction

Ultra-processed foods are industrially manufactured and often contain additives with cosmetic function and non-culinary substances with little to no whole foods. Ultra-processed foods are typically high in sugar, sodium, and saturated fat and low in fiber and other micronutrients [1,2]. The consumption of ultra-processed foods constitutes over half of total daily energy intake in U.S. adults, and this figure has been rising for the past two decades from 53% of total energy intake in 2001 to 57% in 2018 [3,4].

This widespread and continuingly increasing consumption is concerning since existing prospective and meta-analytic studies have consistently demonstrated the link between consuming ultra-processed food and cardiovascular health [4,5,6,7,8]. The high contents of sugar, sodium, saturated fat, and caloric density in ultra-processed food are known to be associated with cardiovascular risk factors including inflammation, hyperglycemia, hypertension, dyslipidemia, and adiposity [9,10,11,12,13]. Food additives for cosmetic function, food substances used in the definition of ultra-processed food (e.g., emulsifiers), extensive heat treatment during production and reheating, and packaging materials are associated with cardiovascular diseases and their risk factors [9,14].

Nevertheless, the current evidence mostly pertains to cardiovascular disease outcomes at the clinical level, and little is known about the relationship between such food and subclinical cardiovascular alterations. When cardiac tissue is damaged, cardiac troponin I (cTnI) and cardiac troponin T (cTnT) are released from cardiomyocytes into the blood [15]. Another potential mechanism for ultra-processed food intake contributing to adverse cardiac health is through cardiac ventricular wall distension. When this happens, pro-B-type natriuretic peptide (proBNP) is produced and is cleaved into the biologically active BNP and the non-biologically active *N*-terminal proBNP (NT-proBNP), which are then secreted into the blood [16,17].

Given the established connection between high ultra-processed food intake and increased risk of cardiovascular disease, alongside the capability of high-sensitivity cTnI (hs-cTnI), hs-cTnT, and NT-proBNP to act as indicators of subclinical myocardial damage and stretch that may help identify individuals at higher cardiovascular risk without prior history of clinical cardiovascular disease [18], this study aimed to investigate the potential association between ultra-processed food consumption and elevated levels of hs-cTnI, hs-cTnT, and NT-proBNP, therefore providing insights on the potential relationship between ultra-processed food and subclinical cardiovascular disease.

## 2. Materials and Methods

### 2.1. Study Population

We conducted cross-sectional analyses using data collected from adult participants (age ≥ 20 years) from two cycles of the National Health and Nutrition Examination Survey (NHANES 2001–2002, 2003–2004) when both cardiac biomarker measurements and classification of foods according to processing level were available. NHANES aims to examine the health and nutrition status of non-institutionalized U.S. civilians. Participants of this survey were selected by a complex, multi-stage probability sampling design [19]. The survey comprises interviews (for demographic, socioeconomic, dietary, and health-related questions) and physical examinations (for medical and physiological measurements and laboratory tests) [19]. Blood specimens were collected by trained and certified medical technologists and phlebotomists at mobile examination centers [20]. The National Center for Health Statistics (NCHS) Ethics Review Board (ERB) was responsible for ensuring the protection of rights and welfare of human participants and that the study conformed to U.S. federal regulations. The collection of data used in this study followed NCHS ERB Protocol #98-12.

From those NHANES 2001–2004 adult participants who had a blood sample collected and provided consent for their serum to be stored for future research (*n* = 10,452), we sequentially excluded those who were pregnant at the time of the physical examination (*n* = 550) and those with prevalent cardiovascular disease (*n* = 1325) defined as a self-reported diagnosis by a health professional of congestive heart failure, coronary heart disease, angina/angina pectoris, heart attack, or stroke. We also excluded participants who had missing dietary intake data (*n* = 974); implausible total energy intake, defined as total energy intake < 600 kcal or >6000 kcal (*n* = 161); or missing data on any cardiac biomarker (*n* = 827). The final analytic sample size was 6615 participants (Supplementary Figure S1).

### 2.2. Dietary Assessment

Dietary intake was assessed using one 24 h dietary recall per person, conducted in person by trained interviewers and coded according to the level of processing using the Nova classification system [21,22]. The Nova classification system categorizes food into four groups based on their industrial processing level and purpose [1]. Nova group 1 consists of unprocessed or minimally processed foods that are subject to little or no processing techniques, such as crushing, freezing, pasteurization, etc., and are without the addition of processed culinary ingredients. Nova group 2 includes processed culinary ingredients that are extracted from group 1 foods or nature and used for culinary preparation, such as oil, sugar, salt, etc. Nova group 3 is comprised of processed foods, which are foods industrially manufactured from group 1 combined with processed culinary ingredients from group 2 using processes such as various cooking methods and preservation techniques. Nova group 4 includes ultra-processed food, which are defined as foods and beverages that have undergone multiple industrial processes and contain non-culinary substances, like high-fructose corn syrup, hydrogenated oil, hydrolyzed protein, and/or additives with cosmetic functions (i.e., flavors, flavor enhancers, and colors). They are typically added to ultra-processed food to enhance texture, palatability, and outlook.

All recorded food and beverages (in food codes) were classified according to mutually exclusive Nova groups and subgroups. For food codes determined to be handmade recipes, the classification was applied to the underlying standard reference (SR) codes obtained from the US Department of Agriculture (USDA) Food and Nutrient Database for Dietary Studies (FNDDS) 2001–2004. The primary exposure was ultra-processed food intake, expressed as the proportion of total daily food intake by weight (%grams), calculated per person as grams of ultra-processed food intake divided by total daily grams of food intake × 100. As a secondary analysis, we used the proportion of total daily energy intake (%kcal) from ultra-processed food (=energy from ultra-processed food/total daily energy intake × 100). The Nova subgroups of ultra-processed food included packaged bread and buns; cake; pie and cookies; ice cream and ice pops; desserts; sugared breakfast cereals; salty snacks; sweet snacks; frozen and shelf-stable plate meals; pizza (ready-to-eat/heat); sandwiches and hamburgers on a bun; French fries and potato products; instant and canned soup, sauces, dressings, and gravies; sugared milk drinks; carbonated soft drinks; sweet beverages; and reconstituted meat and fish products.

### 2.3. Cardiac Biomarker Assessment

Hs-cTnI, hs-cTnT, and NT-proBNP were measured from stored surplus serum samples. All biomarker measurements were conducted during 2018–2020 at the University of Maryland School of Medicine, Baltimore, MD, USA. Abbott ARCHITECT i2000SR (Abbott Park, Illinois) was used to measure hs-cTnI, with coefficients of variation (CVs) between 3.5% and 6.7% [23]. NT-proBNP and hs-cTnT were measured using Roche Cobas e601 (Roche Diagnostics, Basel, Switzerland), with the CVs ranging between 2.7% and 3.1% for NT-proBNP and between 2.0% and 3.1% for hs-cTnT [23,24]. For hs-cTnI and hs-cTnT measurement, the high-sensitivity assays had lower levels of detection, at 1.7 ng/L and 3 ng/L, respectively [23]. The lower and upper limits of detection for NT-proBNP were 5 pg/mL and 35,000 pg/mL, respectively.

The primary outcomes were elevated levels of hs-cTnI, hs-cTnT, and NT-proBNP. Elevated hs-cTnI was defined as >12 ng/L for men and >10 ng/L for women [25]. Hs-TnT ≥ 14 ng/L was considered to be elevated [26]. Elevated NT-proBNP was defined as ≥125 pg/mL for participants < 75 years of age and ≥450 pg/mL for participants ≥75 years of age [27].

### 2.4. Covariates

Age (in years), sex (male or female), race/ethnicity (non-Hispanic white, non-Hispanic black, Mexican, or other), education, smoking status, and physical activity level were obtained via questionnaires, which were administered by trained interviewers. Education was defined as less than high school, high school, or higher than high school. Smoking status was categorized into current smoker, former smoker, and never smoked. Physical activity (yes/no) was defined as active [moderate (at least 10 min per episode, only slight sweating and slight to moderate increase in breathing and heart rate) or vigorous activity (at least 10 min per episode, heavy sweating, and large increase in breathing and heart rate) in past 30 days] or inactive (no moderate or vigorous activity in past 30 days) [28]. Total energy intake (in kcal) was calculated by summing the energy in all food and beverages reported via the 24 h dietary recall.

Body mass index (BMI) and waist circumference (in centimeters) were measured by trained health technicians, and BMI was categorized into normal weight (≥18.5 kg/m^2^ and <25 kg/m^2^), overweight (≥25 kg/m^2^ and <30 kg/m^2^), obese (≥30 kg/m^2^), and underweight (<18.5 kg/m^2^). Blood pressure was measured up to three times by certified physicians and health technologists who received extensive training, and the mean of the readings was used. Hypertension was defined using mean systolic blood pressure ≥130 mmHg, mean diastolic blood pressure ≥80 mmHg, ever being diagnosed with hypertension, or taking anti-hypertensive medication. Diabetes was defined as having hemoglobin A1c ≥6.5%, self-report of a physician diagnosis of diabetes, or currently taking insulin. Estimated glomerular filtration rate (eGFR) was calculated according to the 2021 Chronic Kidney Disease Epidemiology creatinine–cystatin C equation without race and modeled as linear spline terms with two knots at 60 mL/min/1.73 m^2^ and 90 mL/min/1.73 m^2^ [29]. Missing values for covariates were imputed to the mode for categorical variables and to the mean for continuous variables (6 missing for education, 5 missing for smoking, 3 missing for physical activity, 148 missing for BMI, 76 missing for waist circumference, 221 missing for hypertension status, 72 missing for diabetes status, and 31 missing for eGFR). Covariates were selected based on prior knowledge and previous literature.

### 2.5. Statistical Analyses

To account for the complex survey design and to create estimates generalizable to the U.S. adult population, we calculated the survey weight by averaging the 2-year cardiac biomarker weights from the two NHANES cycles, and we incorporated this weight into our study population.

Because individuals with very different energy intakes might not be comparable even if they had a similar percentage of ultra-processed food consumption, we used the residual method to further control for total energy intake. We conducted regression analyses, with total energy intake as the independent variable and the % ultra-processed food consumption as the dependent variable, and the residuals from this regression could provide the estimated effects of ultra-processed food consumption on the cardiac biomarkers independent of total energy intake (i.e., energy-adjusted ultra-processed food consumption).

We examined the socio-demographic, behavioral, clinical, and nutritional characteristics (ultra-processed food subgroups, nutrient intakes) of the energy-adjusted quartiles of ultra-processed food consumption, expressed as a percentage of total daily grams intake and as a percentage of total daily energy intake. Categorical variables were summarized as percentages, and continuous variables were presented in means and standard errors. The distribution of continuous variables was assessed using histograms to evaluate normality. In addition, we also compared the distribution of socio-demographic, behavioral, and clinical characteristics before and after while excluding participants that did not meet the inclusion criteria, as well as before and after while excluding participants with missing or implausible dietary intake data.

We used weighted multivariable logistic regression to investigate the association between energy-adjusted quartiles of ultra-processed food intake and elevation of hs-cTnI, hs-cTnT, and NT-proBNP, after adjusting for potential confounders identified in the literature. The odds ratios and the corresponding 95% confidence intervals were calculated for the elevation of each biomarker, with the first quartile of ultra-processed food consumption being the reference group. Model 1 included demographic characteristics (age, sex, race/ethnicity) and total daily energy intake. Model 2 accounted for the covariates in Model 1 as well as socioeconomic status (education level) and health behaviors (smoking status, physical activity). Model 3 also accounted for health status (BMI status, waist circumference, hypertension status, diabetes status, and eGFR). Model 2 was selected as the main model because it controlled for potential confounders without adjusting for health status variables, which could be potential mediators between ultra-processed food consumption and subclinical cardiovascular disease. For each model, we tested for linear trend across quartiles of ultra-processed food consumption.

We tested for interaction by sex and race on the association between ultra-processed food and cardiac biomarkers using the adjusted Wald test. We conducted a sensitivity analysis using the only available dietary recall for the 2001–2002 cycle and the mean of the two dietary recalls for the 2003–2004 cycle.

All analyses were conducted in Stata version 17.0 (StataCorp, College Station, TX, USA). A two-tail *p* value smaller than 0.05 was considered to be statistically significant. Benjamini–Hochberg false discovery rate (FDR) adjustment was used to account for multiple testing.

## 3. Results

### 3.1. Socio-Demographic Factors, Behaviors, and Health Status

The median percent grams of ultra-processed food intake was 37.0%, ranging from 13% in quartile 1 to 71% in quartile 4 (Table 1). Compared with participants who consumed the least ultra-processed foods (i.e., quartile 1), those who consumed the most ultra-processed foods in %grams (i.e., quartile 4) were younger, more likely to be women and non-Hispanic black, less likely to be non-Hispanic white, and slightly less likely to have received education beyond high school. The total daily energy intake was ~2200 kcal on average across the quartiles. As for health behavior indicators, individuals in quartile 4 were more likely to have never been smokers but slightly less likely to be physically active. As for BMI categories, people in quartile 4 were more likely to have obesity, and they also had a slightly greater waist circumference. However, regarding other clinical characteristics, those in quartile 4 were less likely to have hypertension, diabetes, or eGFR < 60 mL/min/1.73 m^2^.

When ultra-processed food consumption was expressed as %kcal, the median intake was 53%, ranging from 31% in quartile 1 to 75% in quartile 4 (Supplementary Table S1). Participants’ characteristics were generally similar when the ultra-processed food intake was in percent of grams, except that quartile 4 now had the highest proportion of non-Hispanic whites and were no longer more likely to have never smoked.

The sociodemographic, behavioral, and clinical characteristics were similar in the population before excluding any participants, after excluding ineligible participants but retaining those with missing or implausible dietary intake data, and after excluding all ineligible participants (i.e., the study population) (Supplementary Table S2).

### 3.2. Nutritional Characteristics

The intakes of all ultra-processed food subgroups in %grams were higher in quartile 4 of ultra-processed food consumption in %grams (Supplementary Table S3). In quartile 1, bread accounted for the largest proportion of grams of intake among all the ultra-processed food subgroups. From quartile 2 to 4, soft drinks had the highest %grams, and there is a strong dose–response relationship between the consumption of soft drinks and ultra-processed food, where a fourfold increase in soft drink consumption was observed in quartile 4 compared to quartile 1. When ultra-processed food subgroups and quartiles were in %kcal, bread accounted for the largest proportion of energy intake among all the ultra-processed food subgroups consistently across quartiles, followed by soft drinks (Supplementary Table S4). The difference in soft drink consumption across quartiles of ultra-processed food expressed as %kcal was less prominent compared to ultra-processed food expressed as %grams.

Individuals in quartile 4 (in both %grams and %kcal) tended to have a higher intake of added sugar and carbohydrates but a lower intake of fiber, protein, cholesterol, phosphorus, and potassium. On average, intake of total fat, saturated fat, and sodium was similar across quartiles.

### 3.3. Ultra-Processed Food Intake and Cardiac Biomarkers

When ultra-processed food was quantified in percent of total grams of food intake (%grams), no association was found between ultra-processed food consumption and either hs-cTnI or hs-cTnT (Table 2).

**Figure 1:**
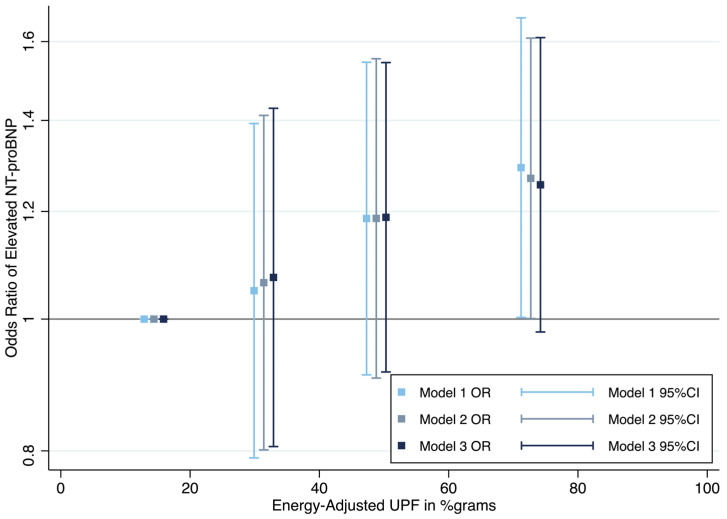
Odds ratio of elevated NT-proBNP across energy-adjusted ultra-processed food percent grams intake (%grams) for Model 1, Model 2, and Model 3. The estimates are plotted at the medians of each quartile (i.e., medians of quartile 1 to 4 of ultra-processed food consumption in %grams). Quartile 1 was the reference group. Model 1 adjusted for age, sex, race category, and total energy intake; Model 2 adjusted for covariates from Model 1 and education category, smoking status, and physical activity status. Model 3 adjusted for covariates from Model 2 and hypertension status, diabetes status, and estimated glomerular filtration rate. NT-proBNP, *N*-terminal prohormone of brain natriuretic peptide; UPF, ultra-processed food; OR, odds Ratio; CI, confidence interval.

However, there were statistically significantly greater odds of elevated NT-proBNP in the highest vs. the lowest quartile of ultra-processed food consumption (Model 1, OR: 1.29, 95% CI: 1.00, 1.67, *p*-trend = 0.02, q-value = 0.16). This association remained significant after further adjusting for education, physical activity status, and smoking status (Model 2, OR: 1.27, 95% CI: 1.00, 1.61, *p*-trend = 0.02, q-value = 0.16). The association between ultra-processed food and NT-proBNP was attenuated and no longer significant after adjusting for clinical covariates including hypertension status, diabetes status, and eGFR (Model 3, OR: 1.26, 95% CI: 0.98, 1.61), though the trend across quartiles remained significant (*p*-trend = 0.03, q-value = 0.21) (Figure 1).

Adding eGFR as a single covariate was primarily responsible for the attenuation in odds ratios from Model 2 to Model 3 (Supplementary Table S5).

When ultra-processed food was expressed as percent of total energy intake (%kcal), no consistent association was observed between ultra-processed food consumption and hs-cTnI, hs-cTnT, or NT-proBNP (Table 3).

No interaction by sex or race on the association between ultra-processed food and cardiac biomarkers was found (*p*-interaction > 0.05) (Supplemental Table S6). The results of sensitivity analyses using the mean of the two recalls for NHANES 2003–2004 were similar to the main results (Supplemental Tables S7 and S8).

## 4. Discussion

In this cross-sectional study of U.S. adult participants, we found that higher ultra-processed food consumption as the proportion of total daily grams intake was associated with elevated NT-proBNP, independent of sociodemographic characteristics, health behaviors, and total daily energy intake. However, no consistent association was observed with any of the cardiac biomarkers (high-sensitivity troponin I, high-sensitivity troponin T, or NT-proBNP) when ultra-processed food consumption was expressed as the proportion of total daily energy intake.

Previous studies have examined the impact of ultra-processed food intake on cardiovascular health. In the NutriNet-Santé cohort, each 10% increment in ultra-processed food intake in proportion of total daily grams intake was associated with a 12% higher risk of cardiovascular disease [5]. In the Framingham Offspring Study, each additional daily serving of ultra-processed food was associated with a 7% greater risk of cardiovascular diseases [6]. In a meta-analysis including more than 1.2 million people, the highest ultra-processed food consumption showed a 17% higher hazard of cardiovascular disease [30]. All these studies, however, focused on hard cardiovascular events. To the best of our knowledge, no study thus far has investigated the association between ultra-processed food consumption and subclinical cardiovascular disease, which can be indicated by the elevation of certain cardiac biomarkers, namely high-sensitivity cardiac troponin I and T and NT-proBNP, in people who appear to be healthy but are at higher risk of clinical cardiovascular disease.

Nevertheless, there have been studies that examined the relationship between dietary factors other than but related to ultra-processed food and NT-proBNP. For example, in a study that assessed the correlation between diet quality and NT-proBNP using data from NHANES 1999–2004, lower intake of sodium and added sugar were associated with lower NT-proBNP by 7.7% and 6.5%, respectively [30]. Many ultra-processed foods like ice cream, cake, sweetened beverages, etc., are usually high in added sugar, and in fact, ultra-processed foods account for nearly 90% of total added sugar intake in the U.S. population [31]. In our study, we found that the intake of added sugar was significantly higher when ultra-processed food consumption was greater regardless of whether the consumption was in %grams or %kcal. Excessive intake of added sugar can prompt a hyperglycemic response, where the rapid rise in blood glucose could potentially lead to inflammation, which in turn may result in an increase in NT-proBNP [32,33]. Based on this evidence, dietary added sugar could be a potential mechanism that contributed to the association found between ultra-processed food consumption and elevated NT-proBNP levels. In addition, high contents of sodium are commonly seen in ultra-processed foods (e.g., savory snacks, instant and canned soup, pizza, etc.). It is possible that the high sodium content in ultra-processed foods may cause water retention, which results in ventricular pressure overload and high flow in blood vessels, thereby leading to increased subclinical cardiac stretch on ventricular walls [17]. The stretch can trigger NT-proBNP secretion from ventricular cardiomyocytes, thus elevating serum NT-proBNP levels [17]. However, in our study, we did not observe higher sodium intake with greater ultra-processed food consumption. A possible explanation for this is reverse causation—participants with the highest consumption of ultra-processed food had the lowest proportion of hypertension, and therefore, it is likely that those who had hypertension tended to limit their intake of ultra-processed foods. In addition, an observational analysis based on the DASH (Dietary Approaches to Stop Hypertension) trial demonstrated that a diet abundant in fruits and vegetables, which are a rich source of dietary fiber and potassium, showed the greatest reduction in NT-proBNP level after the 8-week intervention [34]. In our study, we found that intake of fiber and potassium was lower at higher levels of ultra-processed food consumption. A study that analyzed NHANES data from 1999–2000 found an association between dietary fiber intake and C-reactive protein levels, a marker of inflammation, and inflammation has been shown to increase NT-proBNP levels [33,35]. Moreover, a study has found a direct association between potassium intake and higher NT-proBNP, and there is evidence suggesting a link between low potassium intake and both reactive oxygen species production and endothelial dysfunction, which could in turn lead to cardiac dysfunction [36,37]. Taken together with existing evidence, our study findings suggest that dietary fiber and potassium intake may help explain the observed association between ultra-processed food consumption and elevated NT-proBNP.

Similarly, while studies that specifically looked at the relationship between ultra-processed food and high-sensitivity cardiac troponins are scarce, there is evidence for an association between other dietary patterns or nutrients and cardiac troponins. In the DASH (Dietary Approaches to Stop Hypertension)-Sodium trial, high-sensitivity cardiac troponin I was lower for the DASH diet group (i.e., the more healthful diet group) compared to the control diet (i.e., the typical American diet group), independent of dietary sodium intake [38]. In the Multi-Ethnic Study of Atherosclerosis, there was a cross-sectional association between high sodium intake and high-sensitivity cardiac troponin T [39]. In our study, however, we found no significant association between ultra-processed food consumption and any of the two cardiac troponins, suggesting that subclinical cardiac damage might not be the primary explanation for an association between ultra-processed food consumption and subclinical cardiovascular disease.

Including eGFR in the model attenuated the association between ultra-processed food consumption and NT-proBNP elevation. This attenuation suggests that kidney function may serve as either a confounder or mediator in this association. Greater ultra-processed food intake has been linked to higher risk of chronic kidney disease [40]. Additionally, elevated levels of NT-proBNP have been shown to inversely be correlated with eGFR [41].

In this study, we observed an association between elevated NT-proBNP levels and the consumption of ultra-processed food when it was quantified as %grams of total daily intake, but such association was not seen when it was expressed as %kcal of total daily intake. This discrepancy could be due to the fact that the %grams quantification method may better capture calorie-free/low-calorie beverages and non-nutritional factors like food additives and substances from food packaging [5]. We observed that consumption of soft drinks, including diet beverages, was substantially higher at higher quartiles of ultra-processed food consumption in %grams. The same pattern was observed when expressed as %kcal, but a less substantial difference in soft drink consumption across quartiles of ultra-processed food. As such, the association between ultra-processed food and NT-proBNP levels may be more accurately reflected when ultra-processed food is expressed in %grams rather than %kcal of total daily intake.

Our study has some strengths. First, we conducted this study on a large and nationally representative sample of US adults. Second, the cardiac biomarkers were measured rigorously, and the assays used to measure both cardiac troponins and NT-proBNP have high sensitivity, enabling lower concentrations to be detected, with high reproducibility [23,24]. Third, well-trained interviewers collected data according to standard protocols in NHANES [23,24]. Fourth, we controlled for a comprehensive set of potential confounders.

Our study has several limitations. First, there is a possibility of misclassification by the Nova system we used to identify ultra-processed foods, specifically when the details of the preparation (i.e., homemade or industrially manufactured) or brand name are not known, for example, for a multi-ingredient food [42]. Nevertheless, the Nova food group definitions and examples were sufficient to categorize 90% of the food items reported on a 24 h dietary recall in a national Brazilian dietary survey [42]. Second, we used a single dietary recall per person because a second recall was not collected in the NHANES 2001–2002 cycle, and we chose to maintain consistency across both survey cycles. A single 24 h dietary recall is not adequate to represent one’s routine diet due to the high within-individual day-to-day variability in the types and amount of food consumed and could introduce misclassification and recall bias. Nevertheless, in sensitivity analyses using the mean of two recalls in the 2003–2004 cycle, the associations were similar to those in the main analysis. Third, the cross-sectional design of this study prevents inferences in terms of temporality, and we could not rule out the possibility of reverse causality, whereby participants reduced their intake of high-caloric ultra-processed food because of their health conditions, particularly given the low prevalence of hypertension, diabetes, and eGFR < 60 mL/min/1.73 m^2^ in the highest quartile of ultra-processed food consumption. Forth, social desirability bias could lead to underreporting of ultra-processed food consumption. Fifth, residual confounding may exist, but we adjusted for relevant demographic, behavioral, and clinical covariates, and we applied the residual method in addition to adjusting the model to rigorously control for total energy intake. Sixth, data used in this study were collected in 2001–2004, but dietary patterns have evolved over the past two decades, and thus, this temporal gap may limit the generalizability of our results to the contemporary U.S. population.

The findings of this study have paved the way for further exploration of related topics. Future studies could investigate the association between diet beverages and NT-proBNP elevation given our observation of a difference in the results when ultra-processed food was quantified as %grams versus %kcal. Longitudinal studies could allow for establishing temporality between ultra-processed food consumption and NT-proBNP elevation. Another potential future direction is to investigate the metabolic impact of specific types of additives in ultra-processed food, such as phosphorus and emulsifiers [9,14].

## 5. Conclusions

In conclusion, our study suggests an association between ultra-processed food consumption and NT-proBNP elevation, a biomarker of subclinical cardiac wall stretch, which may predispose individuals to a higher risk of heart failure. No such association was found with elevation of high-sensitivity cardiac troponin I and troponin T, implying that subclinical cardiac damage may not be significantly associated with ultra-processed food consumption. The findings of this study are in line with the recommendations of the 2021 Dietary Guidelines by the American Heart Association, which advocates for a diet higher in minimally processed foods and lower in ultra-processed foods [43]. Given the widespread availability and consumption of ultra-processed food, it is crucial to heighten public awareness about the potential detrimental impact of ultra-processed food on cardiovascular health.

## Figures and Tables

**Table 1:** Characteristics of study population by quartile of the percentage of daily grams of intake from ultra-processed food *.

Characteristics	Total	Quartile 1	Quartile 2	Quartile 3	Quartile 4
Unweighted N ^†^	6615	1772	1703	1609	1531
UPF intake (%grams) median	37.0	12.9	29.9	47.3	71.2
Age, years	44.5 (0.3)	49.2 (0.5)	46.6 (0.6)	43.9 (0.5)	38.2 (0.4)
Female	51.8 (0.6)	48.7 (1.3)	50.2 (1.4)	51.3 (1.6)	56.0 (1.4)
Race/ethnicity					
Non-Hispanic White	72.6 (2.1)	75.7 (2.3)	75.2 (2.2)	71.3 (2.3)	68.2 (3.0)
Non-Hispanic Black	10.5 (1.2)	6.8 (0.9)	7.8 (1.10)	11.0 (1.3)	16.4 (2.0)
Mexican	7.6 (1.1)	6.6 (1.0)	7.9 (1.1)	8.6 (1.3)	7.5 (1.3)
Other ^‡^	9.3 (1.2)	11.0 (1.5)	9.2 (1.2)	9.2 (1.1)	7.9 (1.8)
Education					
Less than high school	16.5 (0.7)	17.1 (1.0)	14.6 (1.0)	17.1 (1.2)	17.2 (1.1)
High school	25.9 (0.8)	23.7 (1.3)	24.1 (1.2)	26.5 (1.7)	29.4 (1.4)
Higher than high school	57.6 (1.2)	59.2 (1.7)	61.3 (1.6)	56.5 (1.8)	53.4 (1.8)
Total energy intake, kcal	2270 (13.5)	2224 (27.5)	2298 (24.3)	2285 (33.7)	2275 (32.5)
Smoking status					
Current smoker	25.2 (1.0)	26.3 (1.1)	23.7 (1.6)	22.1 (1.3)	28.9 (1.6)
Former smoker	23.3 (1.0)	26.2 (1.4)	26.0 (1.7)	24.6 (1.9)	16.7 (1.4)
Never smoked	51.4 (1.2)	47.5 (1.8)	50.3 (1.7)	53.3 (1.5)	54.5 (2.0)
Physically Active	67.7 (1.0)	68.5 (1.8)	69.6 (1.6)	67.3 (1.4)	65.2 (1.8)
BMI					
Underweight	1.7 (0.2)	2.1 (0.4)	1.6 (0.3)	1.0 (0.4)	2.1 (0.5)
Normal weight	34.1 (0.8)	38.7 (1.4)	37.3 (1.7)	31.4 (1.8)	28.9 (0.9)
Overweight	34.4 (0.8)	35.9 (1.6)	36.0 (1.4)	35.2 (1.8)	30.4 (1.1)
Obese	29.8 (0.8)	23.3 (1.4)	25.1 (1.4)	32.3 (1.3)	38.6 (1.1)
Waist Circumference, cm	95.9 (0.3)	94.6 (0.4)	96.0 (0.5)	96.4 (0.5)	96.6 (0.4)
Hypertension	43.4 (0.9)	49.2 (2.0)	43.5 (1.6)	42.8 (1.7)	37.9 (1.4)
Diabetes	7.3 (0.4)	8.4 (0.9)	6.9 (0.6)	7.6 (0.8)	6.5 (0.7)
eGFR < 60 mL/min/1.73 m^2^	2.3 (0.2)	2.6 (0.3)	2.6 (0.4)	2.8 (0.5)	1.1 (0.2)

UPF, ultra-processed food; BMI, body mass index; eGFR, estimated glomerular filtration rate. * Baseline characteristics were reported as weighted mean (SE) for continuous variables and as weighted proportion % (SE) for categorical variables. The SE was calculated from Taylor-linearized variance estimation. The quartiles were calculated based on the residuals (%grams) from regressing ultra-processed food intake (%grams) on total energy intake. ^†^ The distribution of participants across the quartiles was weighted by the survey weights. The reported *N*s are unweighted, but all the estimates are weighted. ^‡^ The ‘Other’ category includes other Hispanics and other races (including multi-racial individuals).

**Table 2:** Odds ratios (95% confidence intervals) of elevated hs-cTnI, hs-cTnT, and NT-proBNP according to quartiles of ultra-processed food intake expressed as percent grams, NHANES 2001–2004 ^†^.

	Categorical UPF Intake (% grams)				*p*-Trend
	Quartile 1 N = 1772	Quartile 2 N = 1703	Quartile 3 N = 1609	Quartile 4 N = 1531	
UPF intake (%grams), median	12.9	29.9	47.3	71.2	
hs-cTnI Elevated ^‡^ (%)	2.2	2.2	1.9	1.7	
Model 1 ^§^	1 [reference]	1.13 (0.65, 1.96)	1.05 (0.61, 1.79)	1.19 (0.70, 2.04)	0.58
Model 2 ^‖^	1 [reference]	1.14 (0.65, 1.99)	1.06 (0.61, 1.84)	1.19 (0.70, 2.02)	0.57
Model 3 ^¶^	1 [reference]	1.16 (0.66, 2.05)	0.97 (0.55, 1.71)	1.20 (0.68, 2.10)	0.68
hs-cTnT Elevated (%)	7.8	7.1	5.4	3.9	
Model 1	1 [reference]	1.20 (0.85, 1.69)	1.16 (0.80, 1.70)	1.39 (0.96, 2.01)	0.11
Model 2	1 [reference]	1.20 (0.85, 1.70)	1.13 (0.78, 1.65)	1.31 (0.90, 1.90)	0.19
Model 3	1 [reference]	1.24 (0.85, 1.82)	0.98 (0.64, 1.49)	1.27 (0.85, 1.91)	0.46
NT-proBNP Elevated (%)	13.9	13.5	12.7	10.2	
Model 1	1 [reference]	1.05 (0.79, 1.39)	1.19 (0.91, 1.54)	1.29 (1.00, 1.67) *	0.02
Model 2	1 [reference]	1.06 (0.80, 1.41)	1.19 (0.90, 1.55)	1.27 (1.00, 1.61) *	0.02
Model 3	1 [reference]	1.07 (0.81, 1.43)	1.19 (0.91, 1.54)	1.26 (0.98, 1.61)	0.03

NHANES, National Health and Nutrition Examination Survey; UPF, ultra-processed food; hs-cTnI, high-sensitivity cardiac troponin I; hs-cTnT, high-sensitivity cardiac troponin T; NT-proBNP, *N*-terminal prohormone of brain natriuretic peptide. ^†^ The quartiles were calculated based on the residuals (%grams) from regressing ultra-processed food intake (%grams) on total energy intake. ^‡^ Elevated indicates the proportion (in %) of study participants for whom the biomarker was elevated. ^§^ Model 1 was adjusted for age, sex, race category, and total energy intake. ^‖^ Model 2 was adjusted for Model 1 covariates plus education category, smoking status, and physical activity status. ^¶^ Model 3 was adjusted Model 2 covariates plus body mass index, waist circumference, hypertension status, diabetes status, and estimated glomerular filtration rate. * *p* < 0.05.

**Table 3:** Odds ratios (95% confidence intervals) for elevated hs-cTnI, hs-cTnT, and NT-proBNP according to quartiles of ultra-processed food intake expressed as percent total energy intake, NHANES 2001–2004 ^†^.

	Categorical UPF Intake (%kcal)				*p*-Trend
	Quartile 1 N = 1771	Quartile 2 N = 1712	Quartile 3 N = 1638	Quartile 4 N = 1494	
UPF intake (%kcal), median	30.9	47.5	60.1	75.2	
hs-cTnI Elevated ^‡^ (%)	2.1	2.0	2.2	1.8	
Model 1 ^§^	1 [reference]	1.01 (0.63, 1.62)	1.11 (0.67, 1.84)	1.03 (0.60, 1.79)	0.81
Model 2 ^‖^	1 [reference]	1.01 (0.63, 1.62)	1.11 (0.67, 1.84)	1.01 (0.58, 1.78)	0.86
Model 3 ^¶^	1 [reference]	1.03 (0.63, 1.71)	1.08 (0.64, 1.81)	0.99 (0.55, 1.77)	0.98
hs-cTnT Elevated (%)	7.1	5.9	6.6	4.7	
Model 1	1 [reference]	0.92 (0.64, 1.31)	1.05 (0.69, 1.58)	0.94 (0.61, 1.42)	0.95
Model 2	1 [reference]	0.91 (0.63, 1.30)	1.04 (0.68, 1.58)	0.90 (0.60, 1.36)	0.82
Model 3	1 [reference]	0.88 (0.62, 1.26)	0.92 (0.60, 1.41)	0.81 (0.52, 1.27)	0.45
NT-proBNP Elevated (%)	11.6	14.4	12.4	11.9	
Model 1	1 [reference]	1.34 (1.03, 1.75) *	1.11 (0.84, 1.46)	1.19 (0.92, 1.53)	0.42
Model 2	1 [reference]	1.36 (1.04, 1.77) *	1.10 (0.85, 1.44)	1.14 (0.89, 1.46)	0.62
Model 3	1 [reference]	1.39 (1.07, 1.81) *	1.09 (0.83, 1.42)	1.13 (0.90, 1.42)	0.78

NHANES, National Health and Nutrition Examination Survey; UPF, ultra-processed food; hs-cTnI, high-sensitivity cardiac troponin I; hs-cTnT, high-sensitivity cardiac troponin T; NT-proBNP, N-terminal prohormone of brain natriuretic peptide. ^†^ The quartiles were calculated based on the residuals (%kcal) from regressing ultra-processed food intake (%kcal) on total energy intake. ^‡^ Elevated indicates the proportion (in %) of study participants for whom the biomarker was elevated. ^§^ Model 1 was adjusted for age, sex, race category, and total energy intake. ^‖^ Model 2 was adjusted for Model 1 covariates plus education category, smoking status, and physical activity status. ^¶^ Model 3 was adjusted Model 2 covariates plus body mass index, waist circumference, hypertension status, diabetes status, and estimated glomerular filtration rate. * *p* < 0.05.

## Data Availability

The data analyzed in this study are publicly available from the National Health and Nutrition Examination Survey (NHANES) and can be accessed at https://wwwn.cdc.gov/nchs/nhanes/default.aspx (accessed on 1 September 2025).

## Supplementary Materials

The following supporting information can be downloaded at: https://www.mdpi.com/article/10.3390/nu17203294/s1, Figure S1: Flow diagram for participant selection; Table S1: Characteristics of US adults according to quartiles of the percentage of daily energy intake from ultra-processed food, NHANES 2001–2004; Table S2: Characteristics of US Adults from NHANES 2001–2004 under different exclusion settings; Table S3: Individual ultra-processed food subgroups intake in %grams of total daily grams intake, as well as daily nutrient intake (added sugar, carbohydrate, total fat, saturated fat, fiber, protein, cholesterol, sodium, phosphorous, and potassium) across quartiles of ultra-processed food intake in %grams. Table S4: Individual ultra-processed food subgroups intake in %kcal of total daily grams intake, as well as daily nutrient intake (added sugar, carbohydrate, total fat, saturated fat, fiber, protein, cholesterol, sodium, phosphorous, and potassium) across quartiles of ultra-processed food intake in %kcal; Table S5: Odds ratios and 95% confidence intervals of elevated NT-proBNP based on quartiles of ultra-processed food intake (%grams) from unadjusted model and adjusted model with each individual covariate of Models 1, 2, and 3; Table S6: Interaction by sex and race on the association between ultra-processed food consumption and cardiac biomarkers; Table S7: Odds ratios (95% confidence intervals) of elevated hs-cTnI, hs-cTnT, and NT-proBNP according to quartiles of ultra-processed food intake in %grams assessed via day-1 dietary recall in NHANES 2001–2002 and the mean of day-1 and day-2 dietary recalls in NHANES 2003–2004; Table S8: Odds ratios (95% confidence intervals) of elevated hs-cTnI, hs-cTnT, and NT-proBNP according to quartiles of ultra-processed food intake in %kcal assessed via day-1 dietary recall in NHANES 2001–2002 and the mean of day-1 and day-2 dietary recalls in NHANES 2003–2004.

## Abbreviations

The following abbreviations are used in this manuscript:

Hs-cTnI	High-sensitivity cardiac troponin I
Hs-cTnT	High-sensitivity cardiac troponin T
NT-proBNP	N-terminal pro-B-type natriuretic peptide
UPF	Ultra-processed food
NHANES	National Health and Nutrition Examination Survey
SR	Standard reference
USDA	US Department of Agriculture
FNDDS	Food and Nutrient Database for Dietary Studies
CV	Coefficient of variation
BMI	Body mass index
eGFR	Estimated glomerular filtration rate
