# Supplementary material for: Ultra-Processed Food Consumption and Subclinical Cardiac Biomarkers: A Cross-Sectional Analysis of U.S. Adults in NHANES 2001–2004
Source: Nutrients. 2025 Oct 20;17(20):3294. doi: 10.3390/nu17203294 (PMC12566985; doi:10.3390/nu17203294)
Supplement: Supplementary file 1 [file nutrients-17-03294-s001.zip › nutrients-3883120-supplementary.pdf]

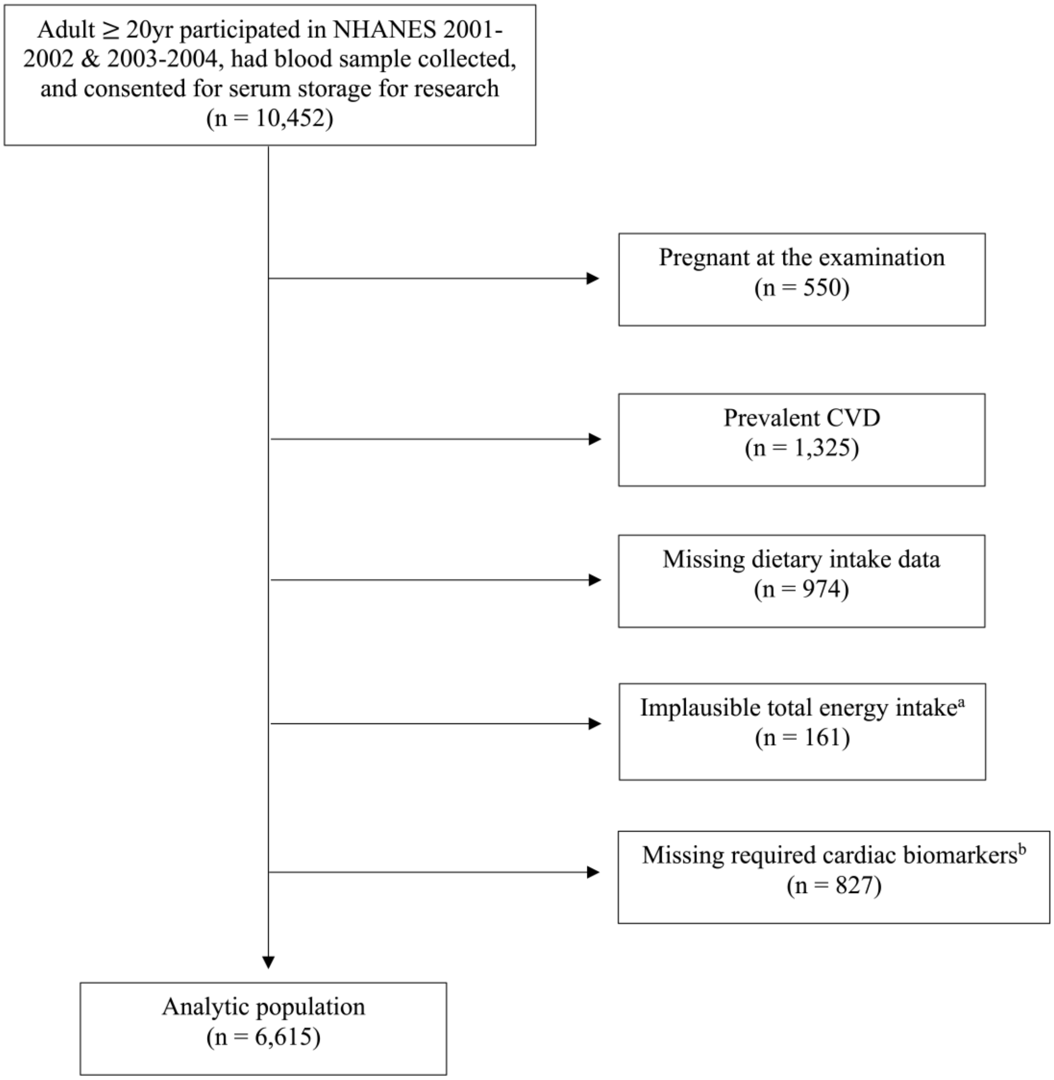

Supplementary Figure S1. Flow diagram for participant selection.

[a] Implausible energy intake means total daily energy intake <600 kcal or >6000 kcal

[b] Participants with missing value of cardiac TnI (n = 759), cardiac TnT (n = 56), and NT-proBNP (n = 12) were excluded sequentially.

CVD, cardiovascular disease.

Supplementary Table S1. Characteristics of US Adults According to Quartiles of the Percentage of Daily Energy Intake from Ultra-Processed Food, NHANES 2001-2004 [*].

**Characteristics**	**Total**	**Quartile 1**	**Quartile 2**	**Quartile 3**	**Quartile 4**
**Unweighted[†] N**	6615	1771	1712	1638	1494
**UPF intake (%kcal) median**	52.5	30.9	47.5	60.1	75.2
**Age, years**	44.5 (0.3)	45.9 (0.4)	45.4 (0.5)	44.5 (0.5)	42.1 (0.5)
**Female**	51.8 (0.6)	49.1 (1.5)	50.8 (1.4)	51.5 (1.2)	55.8 (1.4)
**Race/ethnicity**					
Non-Hispanic White	72.6 (2.1)	67.8 (2.8)	72.8 (2.1)	73.8 (2.1)	75.9 (2.6)
Non-Hispanic Black	10.5 (1.2)	9.2 (1.1)	9.9 (12.6)	10.8 (1.3)	12.0 (1.6)
Mexican	7.6 (1.1)	8.7 (1.3)	8.6 (1.1)	7.8 (1.1)	5.5 (1.1)
Other[‡]	9.3 (1.2)	14.3 (2.0)	8.7 (1.3)	7.6 (1.2)	6.6 (1.4)
**Education**					
Less than high school	16.5 (0.7)	18.5 (1.1)	16.0 (1.3)	15.8 (1.1)	15.6 (1.1)
High school	25.9 (0.8)	21.8 (1.0)	24.8 (1.2)	26.6 (1.0)	30.5 (1.4)
Higher than high school	57.6 (1.2)	59.7 (1.4)	59.2 (1.7)	57.6 (1.3)	53.9 (1.9)
**Total energy intake, kcal**	2270 (13.5)	2246 (29.0)	2294 (30.8)	2302 (30.3)	2239 (24.3)
**Smoking status**					
Current smoker	25.2 (1.0)	26.0 (1.7)	20.8 (1.3)	23.4 (1.5)	30.8 (1.3)
Former smoker	23.3 (1.0)	24.1 (1.6)	24.7 (1.5)	23.7 (1.1)	20.8 (1.1)
Never smoker	51.4 (1.2)	49.9 (1.6)	54.4 (1.9)	52.9 (1.7)	48.4 (1.2)
**Physically Active**	67.7 (1.0)	66.9 (1.6)	68.9 (1.3)	68.6 (1.5)	66.2 (1.7)
**BMI**					
Underweight	1.7 (0.2)	2.4 (0.5)	1.4 (0.3)	1.0 (0.3)	2.0 (0.4)
Normal weight	34.1 (0.8)	35.1 (1.5)	34.8 (1.4)	34.8 (1.4)	31.8 (1.5)
Overweight	34.4 (0.8)	36.7 (1.5)	36.3 (1.6)	32.6 (1.1)	32.0 (1.7)
Obese	29.8 (0.8)	25.8 (1.1)	27.6 (1.6)	31.6 (1.4)	34.2 (1.2)
**Waist Circumference, cm**	95.9 (0.3)	94.5 (0.3)	96.0 (0.4)	96.3 (0.5)	96.9 (0.5)
**Hypertension**	43.4 (0.9)	44.3 (2.0)	43.3 (1.1)	45.1 (1.8)	40.7 (1.8)
**Diabetes**	7.3 (0.4)	8.1 (0.8)	7.1 (0.8)	8.0 (0.7)	6.1 (0.7)
**eGFR < 60 mL/min/1.73m^2**	2.3 (0.2)	2.3 (0.4)	2.3 (0.4)	2.4 (0.5)	2.1 (0.3)

UPF, ultra-processed Food; BMI, body mass index; eGFR, estimated glomerular filtration rate.

[*] Baseline characteristics were reported as weighted mean (SE) for continuous variables and as weighted proportion % (SE) for categorical variables. The SE was calculated from Taylor-linearized variance estimation. The quartiles were calculated based on the residuals (%kcal) from regressing ultra-processed food intake (%kcal) on total energy intake.

[†] The distribution of participants across the quartiles was weighted by the survey weights. The reported *N*s are unweighted, but all the estimates are weighted.

[‡] Other category includes other Hispanics and other race (including multi-racial individuals)

Supplementary Table S2. Characteristics of US Adults from NHANES 2001-2004 under Different Exclusion Settings [*].

Characteristics	Source Population[†]	After Exclusion - Including Missing/Implausible Dietary Intake Data[‡]	After Exclusion – Excluding Missing/Implausible Dietary Intake Data[§]
**Unweighted[‖] N**	21,161	7,086	6,615
**UPF intake (%gram) median [IQR]**	45.4 [26.2, 65.5]	41.4 [20.3, 56.6]	37.0 [20.4, 56.5]
**UPF intake (%kcal) median [IQR]**	57.7 [43.9, 71.4]	52.5 [39.4, 65.6]	52.5 [39.5, 65.5]
**Age, years**	37.2 (0.4)	44.4 (0.3)	44.5 (0.3)
**Female**	51.5 (0.5)	51.7 (1.5)	51.8 (0.6)
**Race/ethnicity**			
Non-Hispanic White	69.3 (2.2)	71.7 (2.2)	72.6 (2.1)
Non-Hispanic Black	12.0 (1.4)	10.9 (1.2)	10.5 (1.2)
Mexican	8.7 (1.2)	7.7 (1.1)	7.6 (1.1)
Other[¶]	10.0 (1.2)	9.7 (1.2)	9.3 (1.2)
**Education**			
Less than high school	18.6 (0.8)	17.1 (0.7)	16.5 (0.7)
High school	26.3 (0.7)	26.1 (0.8)	25.9 (0.8)
Higher than high school	55.1 (1.1)	56.8 (1.1)	57.6 (1.2)
**Total energy intake, kcal**	2206 (12.4)	2278 (15.0)	2270 (13.5)
**Smoking status**			
Current smoker	25.1 (0.9)	25.6 (0.9)	25.2 (1.0)
Former smoker	25.1 (0.9)	23.3 (0.9)	23.3 (1.0)
Never smoker	49.8 (1.1)	51.1 (1.1)	51.4 (1.2)
**Physically Active**	68.5 (0.9)	67.1 (1.0)	67.7 (1.0)
**BMI**			
Underweight	11.1 (0.4)	1.7 (0.2)	1.7 (0.2)
Normal weight	34.9 (0.6)	34.5 (0.8)	34.1 (0.8)
Overweight	28.9 (0.7)	34.3 (0.8)	34.4 (0.8)
Obese	25.1 (0.7)	29.5 (0.8)	29.8 (0.8)
**Waist Circumference, cm**	90.8 (0.3)	96.1 (0.2)	95.9 (0.3)
**Hypertension**	43.7 (0.9)	43.0 (0.9)	43.4 (0.9)
**Diabetes**	7.8 (0.4)	7.3 (0.4)	7.3 (0.4)
**eGFR < 60 mL/min/1.73m^2**	3.1 (0.2)	2.3 (0.2)	2.3 (0.2)

UPF, ultra-processed Food; BMI, body mass index; eGFR, estimated glomerular filtration rate.

[*] Baseline characteristics were reported as weighted mean (SE) for continuous variables and as weighted proportion % (SE) for categorical variables. The SE was calculated from Taylor-linearized variance estimation.

[†] Source population was all participants from NHANES 2000-2004 who consented for serum storage for future research.

[‡] Ineligible participants were excluded but retained those who had missing or implausible dietary intake data.

[§] All ineligible participants including those who had missing or implausible dietary intake data were excluded.

‖ The distribution of participants across the quartiles was weighted by the survey weights. The reported *N*s are unweighted, but all the estimates are weighted.

¶ Other category includes other Hispanics and other race (including multi-racial individuals)

Supplementary Table S3. Individual ultra-processed food subgroups intake in %grams of total daily grams intake, as well as nutrients daily intake (added sugar, carbohydrate, total fat, saturated fat, fiber, protein, cholesterol, sodium, phosphorous, and potassium), across quartiles of ultra-processed food intake in %grams.

**Nutritional factor**	**Quartile 1 N=1772**	**Quartile 2 N=1703**	**Quartile 3 N=1609**	**Quartile 4 N=1531**	**P-trend**
Food subgroups, %grams /day					
Bread	2.70 (0.08)	3.55 (0.10)	3.95 (0.17)	4.08 (0.16)	<0.01
Cakes, cookies, and pies	0.94 (0.06)	1.34 (0.08)	1.44 (0.09)	1.53 (0.11)	<0.01
Ice cream and ice pops	0.61 (0.07)	1.08 (0.11)	1.37 (0.07)	1.44 (0.10)	<0.01
Desserts*	0.27 (0.02)	0.44 (0.04)	0.36 (0.03)	0.31 (0.04)	0.90
Sugared cereals	0.54 (0.04)	0.71 (0.05)	0.63 (0.05)	0.40 (0.04)	<0.01
Salty snacks†	0.58 (0.04)	0.75 (0.03)	0.90 (0.04)	1.05 (0.06)	<0.01
Sweet snacks	0.38 (0.03)	0.52 (0.04)	0.64 (0.08)	0.64 (0.04)	<0.01
Frozen and shelf-stable plate meals	0.19 (0.03)	0.50 (0.07)	0.78 (0.10)	1.36 (0.15)	<0.01
Pizza (ready-to-eat/heat)	0.02 (0.01)	0.06 (0.04)	0.09 (0.04)	0.13 (0.06)	0.05
Sandwiches and hamburgers on bun	0.02 (0.01)	0.02 (0.01)	0.04 (0.02)	0.14 (0.03)	<0.01
French fries and potato products	0.20 (0.03)	0.33 (0.04)	0.56 (0.05)	1.17 (0.10)	<0.01
Instant and canned soup	0.48 (0.05)	0.86 (0.08)	1.02 (0.10)	0.83 (0.12)	<0.01
Sauces, dressings, gravies	1.24 (0.06)	1.71 (0.12)	1.70 (0.09)	1.59 (0.08)	<0.01
Sugared milk drinks‡	0.44 (0.06)	1.10 (0.13)	1.28 (0.15)	1.10 (0.13)	<0.01
Soft drinks, carbonated§	1.85 (0.14)	10.8 (0.46)	21.4 (0.58)	38.5 (0.96)	<0.01
Sweet beverages	0.72 (0.08)	3.72 (0.31)	8.81 (0.52)	15.5 (0.77)	<0.01
Reconstituted meat or fish products	0.80 (0.04)	1.44 (0.07)	1.57 (0.10)	2.26 (0.09)	<0.01
Nutrients, mean (SE)					
Added sugar‖, teaspoon equivalents	10.8 (0.3)	17.6 (0.5)	23.5 (0.6)	33.0 (0.7)	<0.01
Carbohydrate, grams	246.7 (3.8)	272.8 (3.8)	286.5 (3.5)	299.1 (4.1)	<0.01
Total fat, grams	85.5 (1.2)	87.1 (1.0)	85.8 (1.7)	85.2 (1.5)	0.06
Saturated fat, grams	27.4 (0.5)	28.5 (0.4)	28.1 (0.6)	27.8 (0.5)	0.23
Fiber, grams	17.7 (0.4)	17.3 (0.4)	16.0 (0.3)	12.8 (0.3)	<0.01
Protein, grams	90.6 (1.4)	86.6 (1.5)	84.7 (1.5)	75.8 (1.4)	<0.01
Cholesterol, milligrams	329.3 (7.8)	301.0 (8.5)	292.5 (8.3)	263.4 (8.2)	<0.01
Sodium, milligrams	3437.7 (46.2)	3611.8 (46.5)	3588.1 (73.0)	3444.1 (46.2)	0.27
Phosphorous, milligrams	1459.4 (25.2)	1423.3 (17.9)	1359.2 (20.0)	1212.5 (20.2)	<0.01
Potassium, milligrams	3288.3 (47.8)	3000.1 (36.8)	2682.4 (37.4)	2136.0 (38.0)	<0.01

* Including ready-to-eat and dry-mix desserts and sugary products
† Such as crackers, chips, and popcorn
‡ Including flavoured yogurt sweetened with sugar and milkshake.

[§] Including both non-artificially sweetened and artificially sweetened beverages
[||] Added sugar consumption was obtained from the MyPyramid equivalent food database available on U.S. Department of Agriculture (USDA).

Supplementary Table S4. Individual ultra-processed food subgroups intake in %kcal of total daily grams intake, as well as nutrients daily intake (added sugar, carbohydrate, total fat, saturated fat, fiber, protein, cholesterol, sodium, phosphorous, and potassium), across quartiles of ultra-processed food intake in %kcal.

**Nutritional factor**	**Quartile 1 N=1771**	**Quartile 2 N=1712**	**Quartile 3 N=1638**	**Quartile 4 N=1494**	**P-trend**
Food subgroups, %kcal /day					
Bread	6.98 (0.21)	10.51 (0.29)	12.47 (0.26)	13.29 (0.38)	<0.01
Cakes, cookies, and pies	2.01 (0.14)	4.18 (0.25)	5.68 (0.24)	6.99 (0.29)	<0.01
Ice cream and ice pops	1.04 (0.07)	1.94 (0.12)	2.43 (0.21)	3.07 (0.21)	<0.01
Desserts*	0.47 (0.04)	0.83 (0.08)	0.79 (0.08)	0.81 (0.07)	<0.01
Sugared cereals	1.50 (0.11)	2.44 (0.17)	2.58 (0.21)	2.42 (0.17)	<0.01
Salty snacks†	1.83 (0.11)	3.71 (0.17)	4.77 (0.15)	6.29 (0.26)	<0.01
Sweet snacks	0.98 (0.08)	2.14 (0.11)	2.83 (0.17)	3.55 (0.21)	<0.01
Frozen and shelf-stable plate meals	0.21 (0.04)	0.65 (0.08)	1.03 (0.13)	2.59 (0.26)	<0.01
Pizza (ready-to-eat/heat)	0.08 (0.05)	0.11 (0.06)	0.16 (0.09)	0.53 (0.14)	0.02
Sandwiches and hamburgers on bun	0.01 (0.01)	0.06 (0.02)	0.20 (0.07)	0.33 (0.12)	<0.01
French fries and potato products	0.36 (0.06)	0.90 (0.13)	1.65 (0.11)	3.78 (0.24)	<0.01
Instant and canned soup	0.44 (0.06)	0.62 (0.07)	0.58 (0.07)	0.89 (0.09)	<0.01
Sauces, dressings, gravies	2.40 (0.11)	3.41 (0.13)	4.10 (0.15)	4.02 (0.15)	<0.01
Sugared milk drinks‡	0.74 (0.10)	1.02 (0.11)	1.21 (0.10)	1.27 (0.18)	0.02
Soft drinks, carbonated§	2.48 (0.14)	4.20 (0.21)	6.30 (0.34)	9.98 (0.51)	<0.01
Sweet beverages	1.16 (0.08)	2.03 (0.15)	3.12 (0.22)	3.72 (0.32)	<0.01
Reconstituted meat or fish products	1.87 (0.10)	2.99 (0.17)	4.26 (0.17)	7.68 (0.28)	<0.01
Nutrients, mean (SE)					
Added sugar‖, teaspoon equivalents	12.3 (0.3)	18.5 (0.6)	24.1 (0.6)	30.0 (0.7)	<0.01
Carbohydrate, grams	250.0 (3.9)	273.6 (4.9)	288.2 (3.3)	293.3 (3.7)	<0.01
Total fat, grams	83.9 (1.4)	87.1 (1.1)	87.9 (1.5)	84.7 (1.0)	0.70
Saturated fat, grams	27.1 (0.6)	28.7 (0.4)	28.6 (0.5)	27.4 (0.4)	0.49
Fiber, grams	17.6 (0.4)	17.2 (0.4)	16.0 (0.3)	13.2 (0.3)	<0.01
Protein, grams	96.4 (1.7)	89.9 (1.0)	82.6 (1.4)	68.9 (0.9)	<0.01
Cholesterol, milligrams	352.0 (9.1)	324.7 (6.2)	286.8 (7.9)	222.7 (4.7)	<0.01
Sodium, milligrams	3475.2 (55.5)	3583.0 (45.7)	3593.1 (70.5)	3430.3 (45.9)	0.76
Phosphorous, milligrams	1479.2 (31.5)	1435.9 (15.9)	1357.2 (22.5)	1181.9 (14.1)	<0.01
Potassium, milligrams	3190.3 (60.1)	2966.0 (39.1)	2676.5 (44.9)	2273.9 (30.5)	<0.01

* Including ready-to-eat and dry-mix desserts and sugary products

† Such as crackers, chips, and popcorn

‡ Including flavoured yogurt sweetened with sugar and milkshake.

§ Including both non-artificially sweetened and artificially sweetened beverages

‖ Added sugar consumption (in teaspoon equivalents) was obtained from the MyPyramid equivalent food database available on U.S. Department of Agriculture (USDA).

Supplementary Table S5. Odds ratios and 95% confidence intervals of elevated NT-proBNP based on quartiles of ultra-processed food intake (%grams) from unadjusted model and adjusted model with each individual covariate of Model 1, 2, and 3.

	**Quartile 1 N=1772**	**Quartile 2 N=1703**	**Quartile 3 N=1609**	**Quartile 4 N=1531**	**P-trend**
**UPF intake (%grams), median**	12.9	29.9	47.3	71.2	
Unadjusted	1 [reference]	0.96 (0.76, 1.23)	0.89 (0.71, 1.13)	0.70 (0.56, 0.89)	<0.01
Age-adjusted	1 [reference]	1.12 (0.85, 1.47)	1.25 (0.96, 1.62)	1.39 (1.07, 1.80)*	0.01
Sex-adjusted	1 [reference]	0.90 (0.70, 1.15)	0.83 (0.65, 1.07)	0.64 (0.51, 0.81)	<0.01
Race-adjusted	1 [reference]	0.97 (0.76, 1.23)	0.91 (0.72, 1.16)	0.72 (0.58, 0.90)	<0.01
Total energy intake-adjusted	1 [reference]	1.01 (0.80, 1.28)	0.93 (0.73, 1.19)	0.72 (0.57, 0.91)	<0.01
Model 1†	1 [reference]	1.05 (0.79, 1.39)	1.19 (0.91, 1.54)	1.29 (1.00, 1.67)*	0.02
Model 1 + Education	1 [reference]	1.06 (0.79, 1.40)	1.18 (0.91, 1.53)	1.28 (0.99, 1.65)	0.03
Model 1 + smoking status	1 [reference]	1.06 (0.80, 1.41)	1.21 (0.92, 1.59)	1.31 (1.02, 1.68)*	0.01
Model 1 + physical activity status	1 [reference]	1.05 (0.79, 1.40)	1.16 (0.89, 1.52)	1.25 (0.98, 1.60)	0.03
Model 2‡	1 [reference]	1.06 (0.80, 1.41)	1.19 (0.90, 1.55)	1.27 (1.00, 1.61)*	0.02
Model 2 + BMI	1 [reference]	1.09 (0.82, 1.44)	1.24 (0.95, 1.62)	1.33 (1.04, 1.70)*	0.01
Model 2 + waist circumference	1 [reference]	1.07 (0.81, 1.42)	1.21 (0.92, 1.60)	1.32 (1.04, 1.68)*	0.01
Model 2 + hypertension	1 [reference]	1.07 (0.80, 1.42)	1.18 (0.90, 1.55)	1.26 (1.00, 1.60)*	0.02
Model 2 + diabetes	1 [reference]	1.06 (0.80, 1.41)	1.19 (0.90, 1.55)	1.27 (1.00, 1.61)*	0.02
Model 2 + eGFR	1 [reference]	1.05 (0.78, 1.40)	1.13 (0.87, 1.48)	1.19 (0.93, 1.52)	0.08
Model 3§	1 [reference]	1.07 (0.81, 1.43)	1.19 (0.91, 1.54)	1.26 (0.98, 1.61)	0.03

NT-proBNP, N-terminal prohormone of brain natriuretic peptide; UPF, ultra-processed food; BMI, body mass index; eGFR, estimated glomerular filtration rate.

†Model 1 was adjusted for age, sex, race category, total energy intake

‡Model 2 was adjusted for Model 1 covariates plus education category, smoking status, physical activity status

§Model 3 was adjusted Model 2 covariates plus BMI categories, hypertension status, diabetes status, and estimated glomerular filtration rate

*P<0.05

Supplemental Table S6. Interaction by sex and race on the association between ultra-processed food consumption and cardiac biomarkers

	Sex-interaction P-values			Race-interaction P-values		
	**Model 1**	**Model 2**	**Model 3**	**Model 1**	**Model 2**	**Model 3**
**hs-cTnI**	0.84	0.83	0.85	0.16	0.15	0.22
**hs-cTnT**	0.63	0.64	0.49	0.13	0.12	0.29
**NT-proBNP**	0.13	0.18	0.15	0.16	0.13	0.23

Supplemental Table S7. Odds ratios (95% confidence intervals) of elevated hs-cTnI, hs-cTnT, NT-proBNP according to quartiles of ultra-processed food intake in % grams assessed via one dietary recall in NHANES 2001-2002 and the mean of two dietary recalls in NHANES 2003-2004.†

	**Categorical UPF intake (%grams)**				
	**Quartile 1 N=1706**	**Quartile 2 N=1637**	**Quartile 3 N=1560**	**Quartile 4 N=1468**	**P-trend**
**UPF intake (%grams), median**	14.7	30.8	46.9	68.8	
**hs-cTnI**					
**Elevated‡ (%)**	3.3	2.5	2.8	2.9	
**Model 1§**	1 [reference]	0.75 (0.44,1.29)	0.94 (0.59,1.49)	1.19 (0.71,2.01)	0.45
**Model 2‖**	1 [reference]	0.76 (0.44,1.33)	0.95 (0.59,1.52)	1.20 (0.70,2.03)	0.44
**Model 3¶**	1 [reference]	0.79 (0.45,1.36)	0.89 (0.56,1.43)	1.19 (0.70,2.03)	0.54
**hs-cTnT**					
**Elevated(%)**	12.8	11.1	8.8	5.3	
**Model 1**	1 [reference]	0.73 (0.50,1.07)	0.64 (0.44,0.91)	0.43 (0.29,0.63)	<0.01
**Model 2**	1 [reference]	1.02 (0.68,1.53)	1.17 (0.80,1.71)	1.37 (0.87,2.17)	0.15
**Model 3**	1 [reference]	1.01 (0.67,1.53)	1.12 (0.77,1.64)	1.29 (0.80,2.07)	0.26
**NT-proBNP**					
**Elevated(%)**	16.6	15.4	12.7	11.8	
**Model 1**	1 [reference]	1.17 (0.88,1.56)	1.20 (0.91,1.59)	1.51 (1.27,1.81)	<0.01
**Model 2**	1 [reference]	1.19 (0.90,1.58)	1.19 (0.91,1.57)	1.48 (1.25,1.76)	<0.01
**Model 3**	1 [reference]	1.23 (0.92,1.64)	1.22 (0.91,1.62)	1.45 (1.21,1.74)	<0.01

NHANES, National Health and Nutrition Examination Survey; UPF, ultra-processed Food; Hs-cTnI, high-sensitivity cardiac troponin I; Hs-cTnT, high-sensitivity cardiac troponin T; NT-proBNP, N-terminal prohormone of brain natriuretic peptide.

†The quartiles were calculated based on the residuals (%grams) from regressing ultra-processed food intake (%grams) on total energy intake.

‡ Elevated means the proportion (in %) of study participants that had such biomarker elevated

§ Model 1 was adjusted for age, sex, race category, total energy intake

‖ Model 2 was adjusted for Model 1 covariates plus education category, smoking status, physical activity status

¶ Model 3 was adjusted Model 2 covariates plus body mass index, waist circumference, hypertension status, diabetes status, and estimated glomerular filtration rate

*P< 0.05

Supplemental Table S8. Odds ratios (95% confidence intervals) of elevated hs-cTnI, hs-cTnT, NT-proBNP according to quartiles of ultra-processed food intake in %kcal assessed via one dietary recall in NHANES 2001-2002 and the mean of two dietary recalls in NHANES 2003-2004.[†]

	**Categorical UPF intake (%kcal)**				
	**Quartile 1 N=1743**	**Quartile 2 N=1622**	**Quartile 3 N=1557**	**Quartile 4 N=1449**	
**UPF intake (%kcal), median**	33.4	48.2	58.8	72.8	**P-trend**
**hs-cTnI**					
**Elevated[‡] (%)**	3.2	2.2	3.3	3.0	
**Model 1[§]**	1 [reference]	0.79 (0.45,1.39)	1.05 (0.59,1.89)	0.89 (0.51,1.55)	0.95
**Model 2[‖]**	1 [reference]	0.79 (0.45,1.40)	1.03 (0.57,1.87)	0.87 (0.48,1.55)	0.87
**Model 3[¶]**	1 [reference]	0.84 (0.47,1.49)	1.03 (0.57,1.88)	0.84 (0.46,1.53)	0.75
**hs-cTnT**					
**Elevated(%)**	10.4	9.6	10.3	8.0	
**Model 1**	1 [reference]	1.12 (0.80,1.55)	1.13 (0.71,1.82)	1.03 (0.71,1.50)	0.79
**Model 2**	1 [reference]	1.08 (0.78,1.51)	1.10 (0.69,1.75)	0.98 (0.68,1.41)	0.97
**Model 3**	1 [reference]	1.16 (0.81,1.68)	1.08 (0.66,1.77)	0.89 (0.62,1.29)	0.65
**NT-proBNP**					
**Elevated(%)**	13.4	15.8	15.4	12.2	
**Model 1**	1 [reference]	1.34 (0.97,1.84)	1.38 (1.03,1.87)	1.25 (0.95,1.64)	0.07
**Model 2**	1 [reference]	1.33 (0.97,1.83)	1.37 (1.02,1.84)	1.20 (0.92,1.56)	0.13
**Model 3**	1 [reference]	1.39 (1.01,1.93)	1.39 (1.03,1.86)	1.17 (0.89,1.54)	0.22

NHANES, National Health and Nutrition Examination Survey; UPF, ultra-processed Food; Hs-cTnI, high-sensitivity cardiac troponin I; Hs-cTnT, high-sensitivity cardiac troponin T; NT-proBNP, N-terminal prohormone of brain natriuretic peptide.

[†] The quartiles were calculated based on the residuals (%kcal) from regressing ultra-processed food intake (%kcal) on total energy intake.

[‡] Elevated means the proportion (in %) of study participants that had such biomarker elevated

[§] Model 1 was adjusted for age, sex, race category, total energy intake

[‖] Model 2 was adjusted for Model 1 covariates plus education category, smoking status, physical activity status

[¶] Model 3 was adjusted Model 2 covariates plus body mass index, waist circumference, hypertension status, diabetes status, and estimated glomerular filtration rate
